# Interplay of T Helper 17 Cells with CD4^+^CD25^high^ FOXP3^+^ Tregs in Regulation of Allergic Asthma in Pediatric Patients

**DOI:** 10.1155/2014/636238

**Published:** 2014-06-04

**Authors:** Amit Agarwal, Meenu Singh, B. P. Chatterjee, Anil Chauhan, Anuradha Chakraborti

**Affiliations:** ^1^Advanced Pediatric Centre, Post Graduate Institute of Medical Education and Research, Sector 12, Chandigarh 160012, India; ^2^Department of Natural Science, West Bengal University of Technology, Kolkata 700064, India; ^3^Department of Experimental Medicine and Biotechnology, Post Graduate Institute of Medical Education and Research, Chandigarh 160012, India

## Abstract

*Background*. There is evidence that Tregs are important to prevent allergic diseases like asthma but limited literature exists on role of T_H_17 cells in allergic diseases. *Methods*. Fifty children with asthma and respiratory allergy (study group) and twenty healthy children (control group) were recruited in this study. Total IgE levels and pulmonary function tests were assessed. The expression of Tregs and cytokines was determined by flow cytometry. *Results*. The average level of total IgE in study group (316.8 ± 189.8 IU/mL) was significantly higher than controls (50 ± 17.5 IU/mL, *P* < 0.0001). The frequency of T_H_17 cells and culture supernatant level of IL-17 in study group (12.09 ± 8.67 pg/mL) was significantly higher than control group (2.01 ± 1.27 pg/mL, *P* < 0.001). Alternatively, the frequency of FOXP3 level was significantly lower in study group [(49.00 ± 13.47)%] than in control group [(95.91 ± 2.63)%] and CD4^+^CD25^+^FOXP3^+^ to CD4^+^CD25^+^ ratio was also significantly decreased in study group [(6.33 ± 2.18)%] compared to control group [(38.61 ± 11.04)%]. The total serum IgE level is negatively correlated with FOXP3 level (*r* = −0.5273, *P* < 0.0001). The FOXP3 expression is negatively correlated with the IL-17 levels (*r* = −0.5631, *P* < 0.0001) and IL-4 levels (*r* = −0.2836, *P* = 0.0460). *Conclusions*. Imbalance in T_H_17/Tregs, elevated IL-17, and IL-4 response and downregulation of FOXP3 were associated with allergic asthma.

## 1. Introduction

Asthma, characterized by T_H_2 immune response, is a chronic inflammatory disorder, affecting children worldwide [1]. It is now universally accepted that T_H_2 cytokines play a critical role in amplifying asthma [2] whereas T_H_1 cytokines prevent this allergic inflammation [3, 4]. In recent years, it has been shown that the manifestation of asthma in humans is beyond the control of T_H_1 and T_H_2 cells. Some studies have suggested that other T cell subsets like T_H_17 and Treg also play a role in regulating asthma [5]. Treg cells play a key role in the maintenance and tolerance of immune regulation [6] by suppression of T_H_1, T_H_2, T_H_17, and allergen specific IgE. They have also been found to suppress basophils, eosinophils, and mast cells but induce levels of specific IgG4 [7]. Different types of Treg cells are classified as natural and adaptive [8]. Natural Treg cells possess high levels of CD25 (CD25^high^) present on the surface of T cells and the expression of FOXP3 required for the generation and maintenance of their suppressive activity [6, 8, 9]. FOXP3 appears to be a key marker for CD4^+^CD25^+^ T cells and is considered as a master switch for development and function of natural Treg cells [10–13]. Recent studies suggest that Treg cells adopt different mechanisms to suppress immune responses: directly via cell contact and indirectly via reducing the capacity of antigen presentation on antigen presenting cells [14] or via anti-inflammatory cytokines [15, 16]. Some studies have suggested that pulmonary CD4^+^CD25^high^ Tregs are impaired in pediatric asthma [17]. A new subset of CD4^+^ T cells, termed as T_H_17, produces IL-17 [18]. T_H_17 cells are now considered the key mediator in development of asthma [19]. T_H_17 cells enhance both neutrophilic and eosinophilic airway inflammation in mouse model of asthma [20, 21]. T_H_17 cells play a key role in filling the gap between T_H_1 and T_H_2 by secreting IL-17A and IL-17F and also contributing to immunity against certain extracellular bacteria and fungi [22]. IL-17, a proinflammatory cytokine mainly derived from CD4^+^ T cells and also from monocytes, mast cells, macrophages, and neutrophils [23, 24], has been suggested in modulating various inflammatory diseases like asthma in humans [24–26]. T_H_1 and T_H_2 cells as well as T_H_17 differentiation are suppressed by Tregs [27]. However, Treg cells do not suppress T_H_17 cells* in vitro* [28, 29]. Recent evidence indicates that CD4^+^CD25^high^FOXP3^+^ Tregs and T_H_17 cells play an important role in mediating asthma.

*Hypothesis*. The null hypothesis states that T regulatory cells do not play any role in bronchial asthma. We hypothesize that T regulatory cells play a protective role in asthma. T regulatory cells, which regulate the balance between T_H_1 and T_H_2 cells, are downregulated in cases of asthma and allergy.

## 2. Materials and Methods

### 2.1. Subjects

Fifty children with asthma (study group) and twenty healthy children (control group) who were matched for age (in months) (control (88.86 ± 38.67); study group, (85.95 ± 35.55)) attended the Advanced Pediatric Centre in Post Graduate Institute of Medical Education and Research (PGIMER), Chandigarh, and were diagnosed as asthma and were recruited in this study with their informed consent. The sera of age and sex matched nonallergic patients were taken as controls. The Ethics Committee of PGIMER approved this study (Micro/2006/754/8th May 2006).

### 2.2. Methods

The diagnosis of asthma was made by clinical history, physical examination, FEV1 measurement, positive response to bronchodilators, positive skin prick test, and elevated total IgE. The asthma of all patients was under control with inhaled corticosteroids. Blood samples were collected for evaluation of T_H_1, T_H_2, and T_H_17 expression and T_reg_ cells.

### 2.3. Estimation of Total IgE

The total IgE of allergic patients was measured using PATHOZYME immunoglobulin E OD 417 kit. The absorbance was measured at 450 nm after addition of tetramethyl benzidine hydrochloride (TMB) substrate and dilute hydrochloric acid. The concentration of IgE is directly proportional to the color intensity of the test samples. This test was calibrated to WHO 2nd International Reference Preparation 75/502 (1981).

### 2.4. Sample Preparation

Five milliliters of heparinized blood was obtained from 20 healthy subjects and 50 asthmatic patients. For cytokine analysis, plasma was isolated from peripheral blood and stored at −80°C until it was used. Peripheral blood mononuclear cells (PBMC) were isolated from heparinized blood sample by density gradient centrifugation (250 g for 20 minutes at room temperature) using Histopaque (Sigma-Aldrich, Saint Louis, MO, USA).

### 2.5. Flow Cytometric Analysis

The serum levels of cytokines Th1 (IFN-*γ*), Th2 (IL-2, IL-4, IL-6, IL-10, IL-12, and IL-13), and Th17 (IL-17) were assessed using BD CBA flex set. Tests were performed according to manufacturer's instructions (BD Cytometric Bead Array, San Diego, CA). The analysis was carried out using flow cytometry (FACSCanto (Becton Dickinson, Mountain View, CA, USA) with FACS Diva Software).

For analysis of Treg cells, the buffy coat (lymphocytes and monocytes) was separated. The cell pellet washed with PBS (Phosphate Buffer Saline) was centrifuged at 200 g for 15 minutes. PBMCs were cultured in a petri dish containing 5% CO_2_ at 37°C for one and half hour. Surface phenotyping (CD4 and CD25) of the cells (peripheral blood lymphocytes) and intracellular phenotyping (FOXP3) were performed by staining, paraformaldehyde fixation, and permeabilization according to the manufacturer's instructions (BD biosciences San Diego, CA). PBMCs were determined using forward and side scatter properties based on size and granularity by FACSCanto (Becton Dickinson, Mountain View, CA, USA) with FACS Diva Software. The following mAbs (BD biosciences) were used: APC antihuman CD4, PE-Cy antihuman CD25, and PE antihuman FOXP3. To correct nonspecific binding, matched isotype controls were used.

### 2.6. Statistical Analysis

Data were analyzed with SPSS (v16.0; SPSS Inc, Chicago, IL, USA) and Graphpad prism (v5.0; Graphpad software Inc, Le Jolla, CA, USA). The mean values and their internal differentiation with standard deviations were calculated. The spearman's *r* rank correlation coefficients were used to evaluate relationship between variables. When assessing the flow cytometric data, Student's* t*-test was used. *P* values <0.05 were considered statistically significant.

## 3. Results

**Figure 1:**
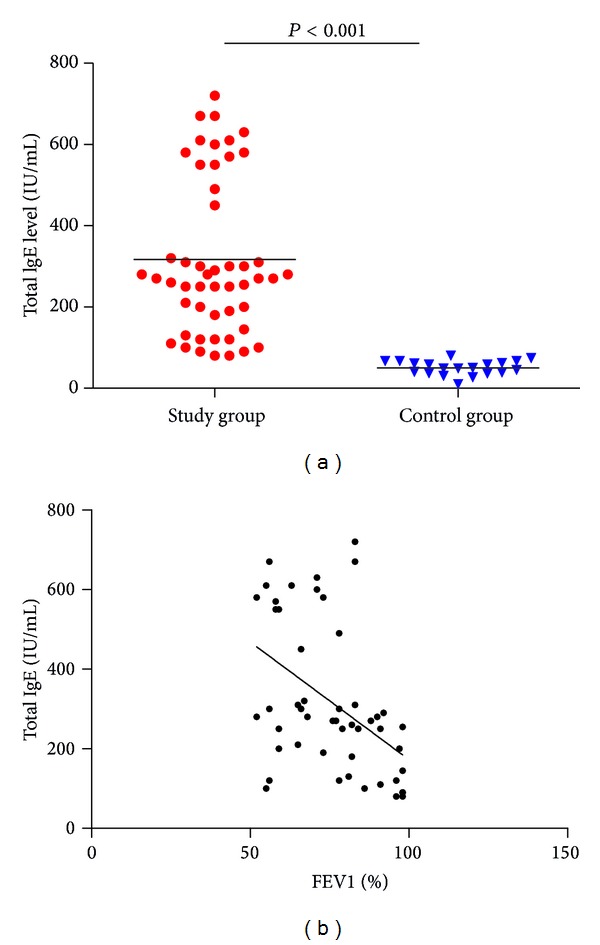
(a) Total IgE level in study and control group. (b) Correlation between total IgE and % FEV1 levels in study group.

Total IgE and FEV1 levels were tested in all children diagnosed with asthma. The difference between IgE levels in study and control group was analyzed using Graphpad Prism software. The nonparametric Student's* t*-test was applied between study group and control group for total IgE level. The average level of total IgE in study group (316.8 ± 189.8 IU/mL, range 80–720 IU/mL) was significantly higher than in control group (50.3 ± 17.5 IU/mL, range 10–80 IU/mL, *P* < 0.001) (Figure 1(a)). FEV1 (% predicted) was significantly lower in study group (75.36 ± 14.45) compared to control group (102.3 ± 8.97, *P* < 0.0001) (Table 1). Total serum IgE and FEV1 levels were also analyzed for their correlation studies. This was analyzed using Spearman's correlation coefficient in study group only. There was a negative correlation between total IgE and FEV1% levels (*r* = −0.4820, *P* = 0.0004**)** (Figure 1(b)).

### 3.1. Expression of Cytokines in Asthmatic Children

**Figure 2:**
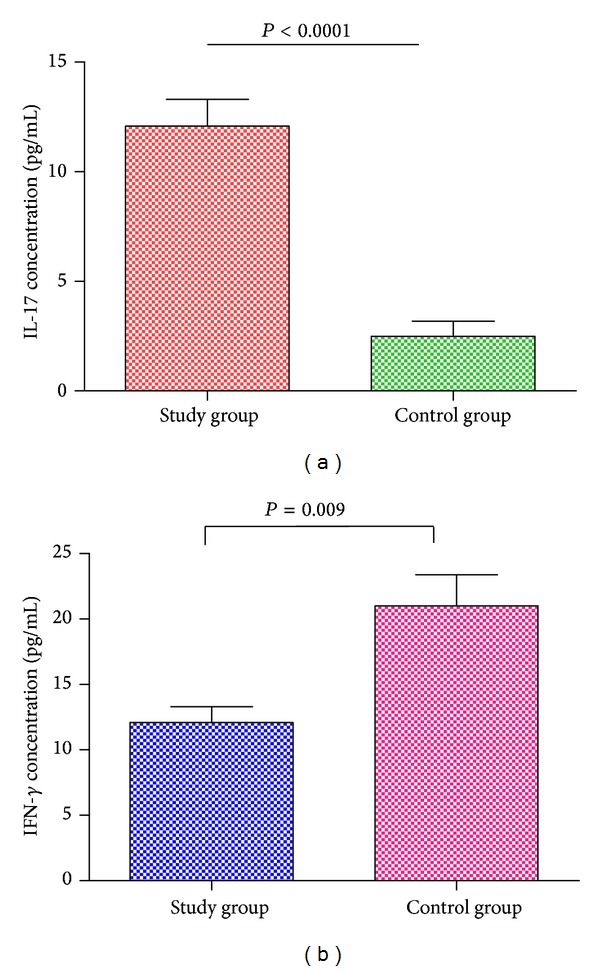
(a) IL-17 and (b) IFN-*γ* expression in study and control.

Levels of cytokines IL-2, IL-4, IL-6, IL-10, IL-12, IL-13, IFN-*γ*, and IL-17 in sera were expressed as mean ± standard deviation. The average level of IL-17 expression in study group (12.09 ± 8.67 pg/mL) was significantly higher than the corresponding values in control group (2.01 ± 1.27 pg/mL, *P* < 0.0001) (Figure 2(a)) but values of IFN-*γ* were significantly lower in study group (12.08 ± 8.67 pg/mL) compared to control group (21.00 ± 7.53 pg/mL, *P* = 0.009) (Figure 2(b)). No significant difference was observed between study and control group for other cytokines (IL-2: 20.78 ± 9.22 pg/mL versus 18.93 ± 13.73 pg/mL (*P* = 0.51); IL-4: 21.88 ± 10.35 pg/mL versus 19.79 ± 12.38 pg/mL (*P* = 0.47); IL-6: 18.17 ± 10.49 pg/mL versus 15.11 ± 9.79 pg/mL (*P* = 0.08); IL-10: 22.82 ± 19.16 pg/mL versus 18.62 ± 5.31 pg/mL (*P* = 0.35); IL-12: 17.58 ± 9.27 pg/mL versus 16.94 ± 11.00 (*P* = 0.52); IL-13: 34.55 ± 17.51 pg/mL versus 29.39 ± 10.12 pg/mL (*P* = 0.40)) (Table 2).

The Student's* t*-test was done to analyze IL-17 in control and study group. The results depict a significant difference between the two groups (*P* < 0.0001). Similarly, there was also significant difference between study group and control group for IFN-*γ* (*P* = 0.009).

**Figure 3:**
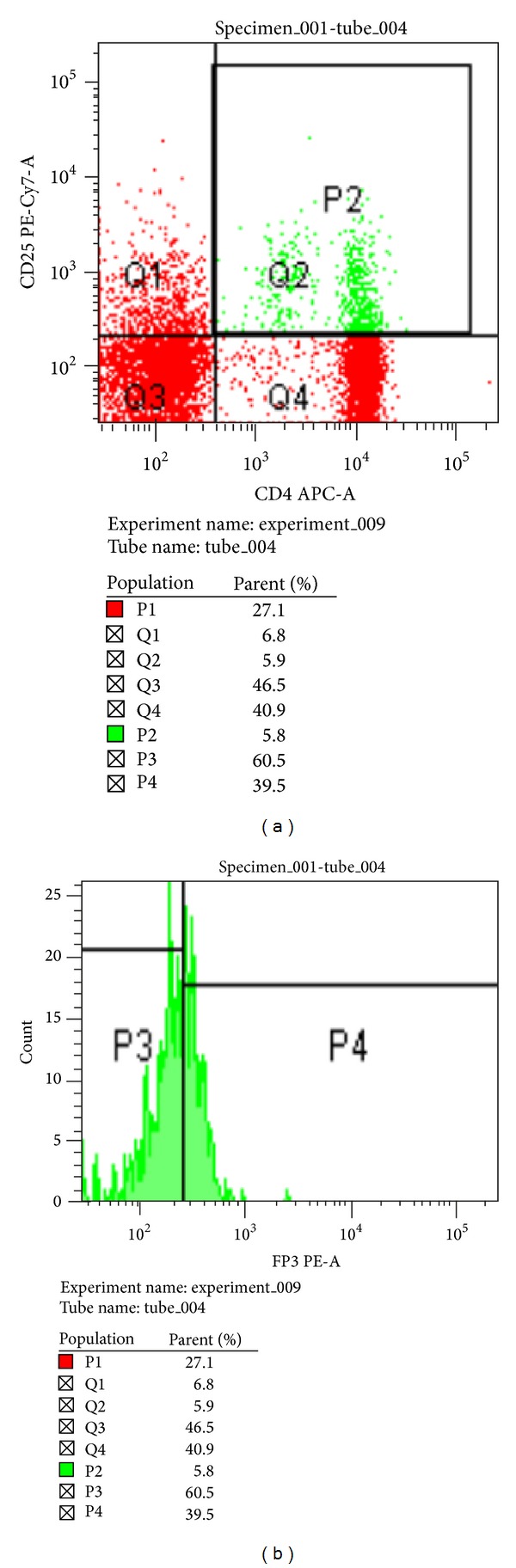
The expression level of CD4^+^CD25^+^ and FOXP3 was examined by flow cytometry in control group. (a) Representation plots of CD4^+^CD25^+^ cells (b) and FOXP3 expression in control group.

**Figure 4:**
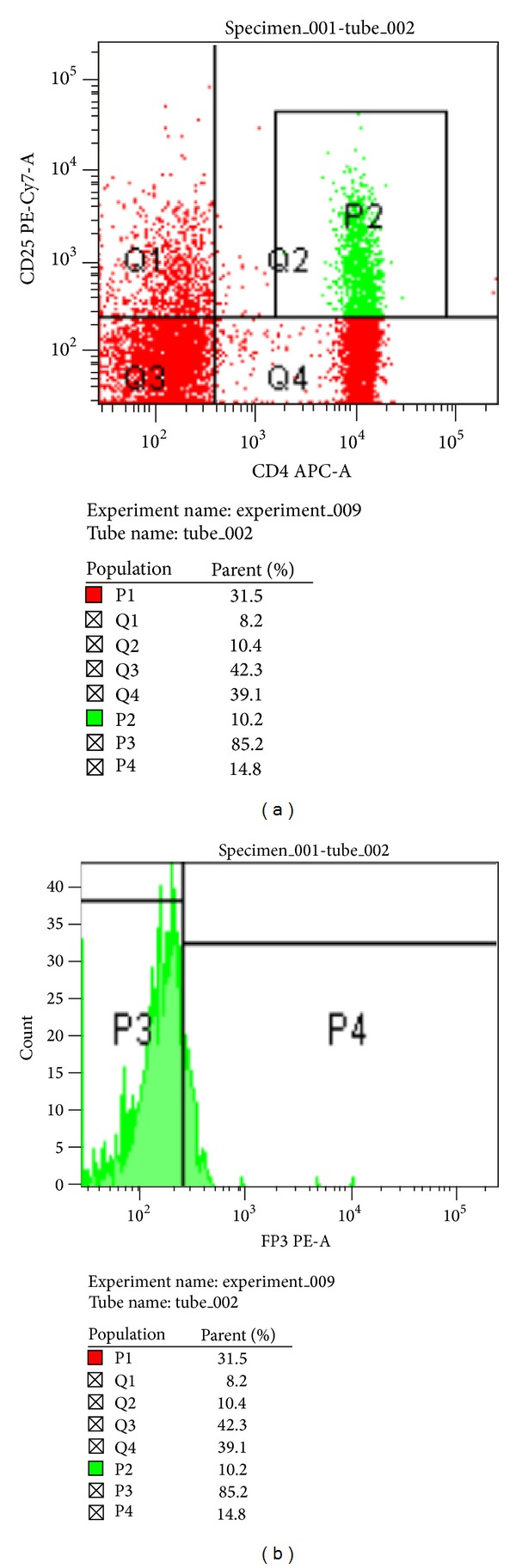
The expression level of CD4^+^CD25^+^ and FOXP3 was examined by flow cytometry in asthmatic group. (a) Representation plots of CD4^+^CD25^+^ cells (b) and FOXP3 expression in study group.

**Figure 5:**
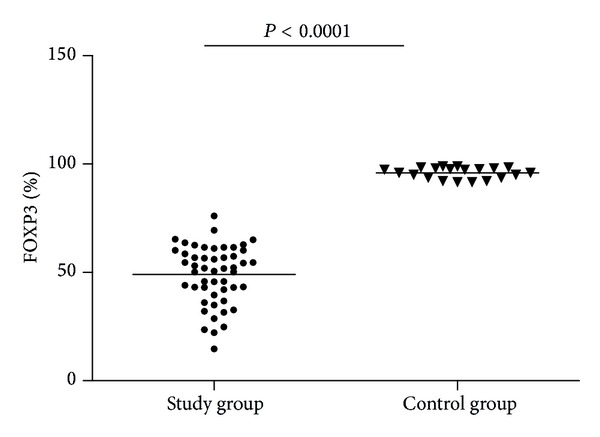
FOXP3% in study and control group.

Flow cytometric analysis of FOXP3 was performed in CD4^+^CD25^+^ cells for both control (*n* = 20) (Figure 3) and study group (*n* = 50) (Figure 4). The percentages of FOXP3 expression were significantly lower in study group ((49.00 ± 13.47)%) than in control group ((95.91 ± 2.63)%, *P* < 0.0001) (Figure 5).

**Figure 6:**
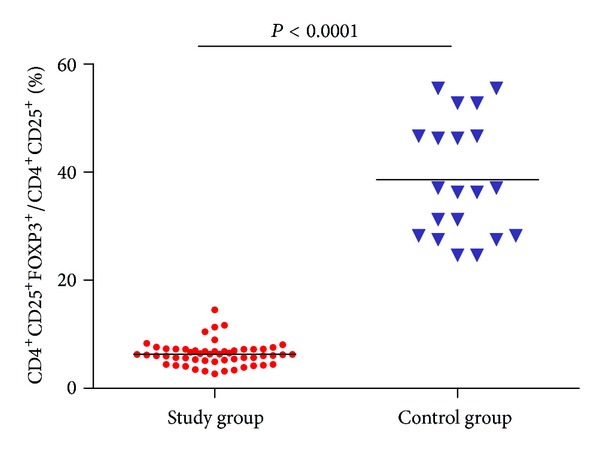
CD4^+^CD25^+^FOXP3^+^/CD4^+^CD25^+^ (%) in study and control group.

A further analysis was done to calculate CD4^+^CD25^+^FOXP3^+^ to CD4^+^CD25^+^ ratio, which was significantly decreased in study group ((6.33 ± 2.18)%) compared to control group ((38.61 ± 11.04)%, *P* < 0.0001) (Figure 6).

### 3.2. Correlation Analysis

#### 3.2.1. Relationship of Total IgE and FOXP3 Expression

**Figure 7:**
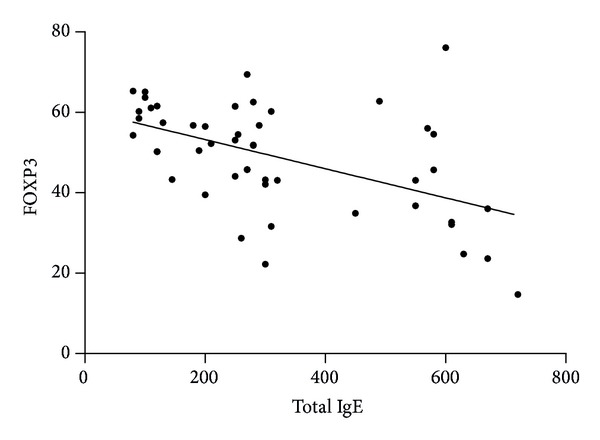
Correlation between FOXP3 and Total IgE.

There was a significant negative correlation between %FOXP3/CD4^+^CD25^high^ and total IgE level (*r* = −0.5273, *P* < 0.0001) (Figure 7).

#### 3.2.2. Interaction between FOXP3 Expression and Level of IL-17 and IL-4

**Figure 8:**
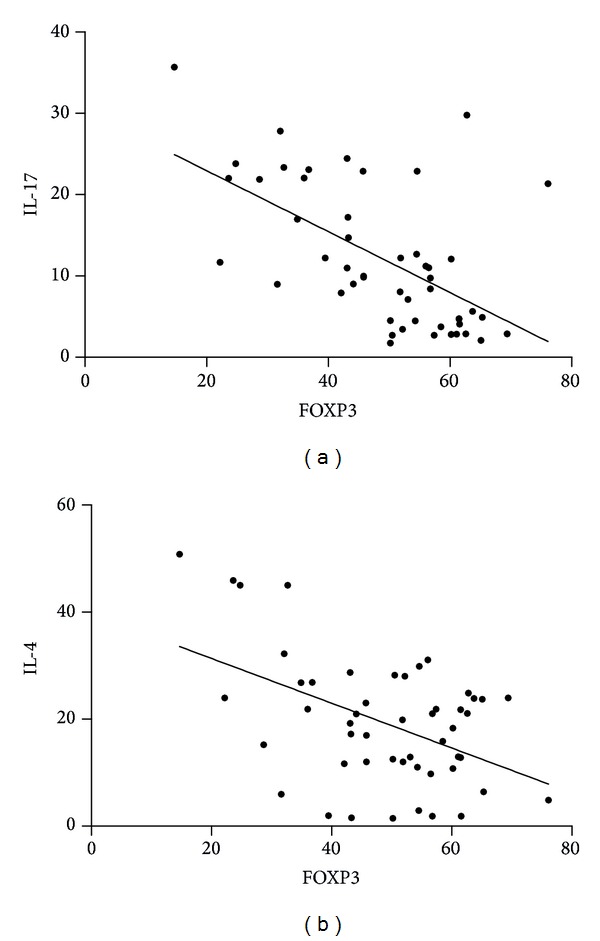
Correlation (a) between FOXP3 and IL-17 and (b) between FOXP3 and IL-4.

FOXP3 percentage and IL-17 level had a significant inverse correlation with each other (*r* = −0.5631, *P* < 0.0001) (Figure 8(a)). There was also a significant correlation between %FOXP3 and IL-4 (*r* = −0.2836, *P* = 0.0460) (Figure 8(b)).

## 4. Discussion

The present work demonstrates the relationship between T_H_17 and Treg cells. It is universally accepted that total IgE level is directly correlated with allergy and asthma. In our study, the average level of total IgE was significantly higher in children with bronchial asthma compared to healthy subjects. On the basis of available studies, we had hypothesized that Treg cells would be associated with lower levels of allergy markers such as IgE and T_H_2 cytokines. Most studies of Treg activity come from immunotherapy studies in allergic diseases [30]. FOXP3 transcription factor has been shown as a key regulator for development of Treg cells and is expressed by these cells [10, 11, 31]. In our study, we found that FOXP3 level is significantly lower in study group compared to control group. Furthermore, there was a negative correlation between total IgE and FOXP3 expression. In this study, we also demonstrated a T_H_17/Treg cytokine profile in study group. Studies have suggested that asthma is associated with chronic and recurrent inflammation [32]. T_H_2 cells are associated directly with inflammation whereas T_H_17 cells behave primarily as proinflammatory markers [33]. Studies suggested that transcription factors and cytokines are involved in generation, differentiation, and expansion of T_H_17 cells. The interaction between T_H_17 cells and Tregs in various inflammatory diseases needs to be further defined [34]. The knowledge of suppressive activity of Treg cells in atopic disease is still contradictory and limited. This study supports the notion that function of Tregs is altered or impaired in allergic patients compared to healthy individuals [13, 17, 35–40]. However, there are some studies that have shown results going the opposite way [41–43]. These alterations may be related to different allergic diseases, different environmental influences, and differences in methodology for identification of cell markers that are used in proper identification of Tregs. In our study, we found significantly higher IL-17 level in asthmatic patients compared to controls. Previous studies showed that IL-17 is elevated in sputum samples of patients with asthma compared to healthy controls [44–46]. Another study showed that patients with asthma had elevated IL-17 levels in serum compared with control subjects [47]. It has been suggested that IL-17 plays an important role in inflammatory and autoimmune diseases [48]. In patients with asthma, IL-17 level was significantly increased and T cell population was skewed toward T_H_17 phenotype. Thus, there is a correlation of increase in IL-17 levels in patients with asthma coupled with a significant decrease in transcription factor FOXP3 Treg level when compared to controls.

We could not find a study in children with asthma reporting the relationship of T_H_17 with Treg response in the milieu of T_H_2 activity. These results show that there is a correlation between FOXP3 and IL-17 level and also a functional imbalance in T_H_17/Treg in children with asthma. In this study, we demonstrated that IL-17 and FOXP3 are reciprocally interconnected with each other. It has already been shown that CD4^+^CD25^+^FOXP3^+^ play a protective role in autoimmune disease [12]. We found that the suppressive activity of CD4^+^CD25^high^ T cells was variable, which is already reported in previous studies [49, 50]. FOXP3 transcription factor plays a key role in regulation and development of CD4^+^CD25^+^ T cells and is expressed by these cells [10, 11, 31]. Our study also shows that there is a significant negative correlation between IL-4 and FOXP3.

In conclusion, the present study demonstrates that there is an imbalance between T_H_17 and Tregs associated with asthma, which may play a potential role in development of asthma. Our study also shows inverse correlation between IL-17 and FOXP3. Future studies are needed to clarify these findings.

## Figures and Tables

**Table 1:** Patients characteristics.

	Study group	Control group
Total subjects	50	20
Age (months) (mean ± SD)	88.86 ± 38.67	85.95 ± 35.55
Male; *n* (%)	40 (80%)	14 (70%)
Total IgE (IU/mL) (mean ± SD)	316.8 ± 189.8	50.3 ± 17.5
FEV_1_ % (mean ± SD)	75.36 ± 14.45	102.3 ± 8.97

**Table 2:** Cytokine expression in study group with SPT positive for one or more food allergen and control group.

Cytokines	Study group* *N* = 50	Control group* *N* = 20	*P* value
IFN-*γ*	12.46 ± 8.88	21.00 ± 7.53	*P* = *0.009 *
IL-2	18.93 ± 13.73	20.78 ± 9.22	*P* = *0.51 *
IL-4	19.79 ± 12.38	21.88 ± 10.35	*P* = *0.47 *
IL-6	15.11 ± 9.79	18.17 ± 10.49	*P* = *0.08 *
IL-10	22.82 ± 19.16	18.62 ± 5.31	*P* = *0.35 *
IL-12p7	16.94 ± 11.00	17.58 ± 9.27	*P* = *0.52 *
IL-13	34.55 ± 17.51	29.39 ± 10.12	*P* = *0.40 *
IL-17A	12.09 ± 8.67	2.01 ± 1.27	*P* < *0.0001 *

*Values are expressed in Mean ± SD pg/mL.

## Abbreviations

FOXP3: Forkhead box P3
IL: Interleukin
Treg: Regulatory T cell
FACS: Fluorescence-activated cell sorting
IgE: Immunoglobin E
FEV1: Forced expiratory volume 1
HRP: Horseradish peroxidase
mAbs: Monoclonal antibodies
PBMC: Peripheral blood mononuclear cell.
